# Dissemination of *Mycobacterium tuberculosis* is associated to a *SIGLEC1* null variant that limits antigen exchange via trafficking extracellular vesicles

**DOI:** 10.1002/jev2.12046

**Published:** 2021-01-14

**Authors:** Susana Benet, Cristina Gálvez, Francis Drobniewski, Irina Kontsevaya, Lilibeth Arias, Marta Monguió‐Tortajada, Itziar Erkizia, Victor Urrea, Ruo‐Yan Ong, Marina Luquin, Maeva Dupont, Jakub Chojnacki, Judith Dalmau, Paula Cardona, Olivier Neyrolles, Geanncarlo Lugo‐Villarino, Christel Vérollet, Esther Julián, Hansjakob Furrer, Huldrych F. Günthard, Paul R. Crocker, Gustavo Tapia, Francesc E. Borràs, Jacques Fellay, Paul J. McLaren, Amalio Telenti, Pere‐Joan Cardona, Bonaventura Clotet, Cristina Vilaplana, Javier Martinez‐Picado, Nuria Izquierdo‐Useros

**Affiliations:** ^1^ Department of Retrovirology IrsiCaixa AIDS Research Institute Badalona Spain; ^2^ Department of Retrovirology Universitat Autònoma de Barcelona Cerdanyola del Vallès Spain; ^3^ Department of Retrovirology Imperial College London UK; ^4^ Department of Retrovirology Research Center Borstel, Borstel Germany; ^5^ Department of Retrovirology N.V. Postnikov Samara Region Clinical Tuberculosis Dispensary Samara Russia; ^6^ Experimental Tuberculosis Unit (UTE) Germans Trias i Pujol Health Science Research Institute Can Ruti Campus Badalona Spain; ^7^ Departament de Genètica i de Microbiologia Facultat de Biociències Universitat Autònoma de Barcelona Cerdanyola del Vallès Spain; ^8^ Centro de Investigación Biomédica en Red de Enfermedades Respiratorias (CIBERES) Madrid Spain; ^9^ REMAR‐IVECAT Group Germans Trias i Pujol Health Science Research Institute Can Ruti Campus Badalona Spain; ^10^ ICREC Research Program Germans Trias i Pujol Health Science Research Institute Can Ruti Campus Badalona Spain; ^11^ Department of Cell Biology Physiology and Immunology Universitat Autònoma de Barcelona Cerdanyola del Vallès Spain; ^12^ Division of Cell Signalling and Immunology University of Dundee Dundee UK; ^13^ Institut de Pharmacologie et Biologie Structurale IPBS CNRS UPS Université de Toulouse Toulouse France; ^14^ International associated laboratory (LIA) CNRS “IM‐TB/HIV” (1167) France and Buenos Aires Toulouse Argentina; ^15^ Department of Infectious Diseases Bern University Hospital University of Bern Bern Switzerland; ^16^ Division of Infectious Diseases and Hospital Epidemiology University Hospital Zurich Zurich Switzerland; ^17^ Institute of Medical Virology University of Zurich Zurich Switzerland; ^18^ Pathology Department Hospital Universitario Germans Trias i Pujol Badalona Spain; ^19^ Germans Trias i Pujol Research Institute (IGTP) Can Ruti Campus Badalona Spain; ^20^ Nephrology Department Germans Trias i Pujol University Hospital Badalona Spain; ^21^ School of Life Sciences École Polytechnique Fédérale de Lausanne Lausanne Switzerland; ^22^ Swiss Institute of Bioinformatics Lausanne Switzerland; ^23^ Precision Medicine Unit Lausanne University Hospital and University of Lausanne Lausanne Switzerland; ^24^ JC Wilt Infectious Diseases Research Centre Public Health Agency of Canada Winnipeg Manitoba Canada; ^25^ Department of Medical Microbiology and Infectious Diseases University of Manitoba Winnipeg Manitoba Canada; ^26^ The Scripps Research Institute La Jolla California USA; ^27^ AIDS and Related Illnesses Centre for Health and Social Care Research (CESS) Faculty of Medicine University of Vic ‐ Central University of Catalonia (UVic ‐ UCC) Vic Spain; ^28^ Catalan Institution for Research and Advanced Studies (ICREA) Barcelona Spain

**Keywords:** Extracellular vesicles, HIV‐1, Mtb, Siglec‐1

## Abstract

The identification of individuals with null alleles enables studying how the loss of gene function affects infection. We previously described a non‐functional variant in *SIGLEC1*, which encodes the myeloid‐cell receptor Siglec‐1/CD169 implicated in HIV‐1 cell‐to‐cell transmission. Here we report a significant association between the *SIGLEC1* null variant and extrapulmonary dissemination of *Mycobacterium tuberculosis* (Mtb) in two clinical cohorts comprising 6,256 individuals. Local spread of bacteria within the lung is apparent in Mtb‐infected Siglec‐1 knockout mice which, despite having similar bacterial load, developed more extensive lesions compared to wild type mice. We find that Siglec‐1 is necessary to induce antigen presentation through extracellular vesicle uptake. We postulate that lack of Siglec‐1 delays the onset of protective immunity against Mtb by limiting antigen exchange via extracellular vesicles, allowing for an early local spread of mycobacteria that increases the risk for extrapulmonary dissemination.

## 1 INTRODUCTION

The phenotypic consequences of genetic null variants that result in loss‐of‐function illuminate the biological role of human proteins *in vivo*. Naturally occurring human null individuals that reduce or enhance infection‐associated diseases can provide *in vivo* validation of the safety and efficacy of future therapeutic strategies, and allow the rational identification of novel targets (Deboever et al., 2018). Yet, despite its vast clinical potential, studying the effects of rare genetic null variants in the context of natural infections remains challenging (Martinez‐Picado et al., 2017; Quintana‐Murci et al., 2007).

We have previously identified HIV‐1 infected individuals harbouring a truncating or null variant in the *SIGLEC1* gene, which abrogates the expression of the Siglec‐1 (CD169) protein (Martinez‐Picado et al., 2016). Siglec‐1 is a myeloid‐cell surface receptor that mediates HIV‐1 capture, transfer and infection of bystander CD4^+^ T cells (Izquierdo‐Useros et al., 2012; Puryear et al., 2013). The null *SIGLEC1* variant confers an early stop codon at amino acid position 88 (Glu88Ter*; rs150358287) and is found at higher allele frequency in individuals of European or South Asian ancestry (>1.1%), while it is rare or absent in African and East Asian populations (<0.5%). Functional assays have previously shown that in heterozygosis, the Glu88Ter* or null variant reduces Siglec‐1 expression by half and therefore causes haploinsufficiency, while in homozygosis it abrogates the expression of the receptor (Martinez‐Picado et al., 2016). Prior analysis of more than 4000 HIV‐1‐infected individuals identified homozygous and heterozygous subjects, whose cells were functionally null or partially defective for Siglec‐1‐mediated capture of HIV‐1 and its transmission *ex vivo* (Martinez‐Picado et al., 2016). This allowed us to investigate the *in vivo* contribution of Siglec‐1 to HIV‐1 disease progression.

Siglec‐1 binds HIV‐1 through recognition of sialylated gangliosides that are anchored on the viral lipid membrane (Izquierdo‐Useros et al., 2012; Puryear et al., 2012), and this mechanism is exploited by several other viruses that also incorporate sialylated ligands (Akiyama et al., 2014; Erikson et al., 2015; Kijewski et al., 2016; Perez‐Zsolt et al., 2019). Yet, the primary role of Siglec‐1 is to favour immune surveillance and promote pathogen containment. Recognition of sialylated ligands on immune cells modulates Siglec‐1 capacity to induce T cell responses (Macauley et al., 2014), via interactions with either antigen presenting cells or specific T cell populations (Kidder et al., 2013; Van Dinther et al., 2018). Moreover, Siglec‐1 interacts with sialylated bacteria to promote host defence and pathogen clearance (Chang et al., 2014; Heikema et al., 2010; Jones et al., 2003; Klaas et al., 2012). This has been reported for bacteria containing sialylated lipopolysaccharides such as *Campylobacter jejuni*, group B Streptococcus or certain meningococcus (Chang et al., 2014; Heikema et al., 2010; Jones et al., 2003; Klaas et al., 2012). In addition, Siglec‐1 also captures sialylated extracellular vesicles (Díaz‐Varela et al., 2018; Izquierdo‐Useros et al., 2012; Saunderson et al., 2013) secreted by antigen‐presenting cells interacting with pathogens or by productively infected cells (Yáñez‐Mó et al., 2015). Transfer of antigens between immune cells via extracellular vesicles amplifies the initiation of immunity (Théry et al., 2009), both through direct MHC recycling but also promoting cross‐presentation. In turn, this initial boost of antigen transmission mediated by extracellular vesicles between antigen presenting cells is paramount to mount early and effective responses and contain pathogen invasion.

Although Siglec‐1 can modulate immune responses to infection, sialylated viruses like HIV‐1 subvert Siglec‐1‐dependent interactions to hijack antigen presenting cells (Macauley et al., 2014; Perez‐Zsolt et al., 2019). In the context of an HIV‐1 infection, we hypothesized that co‐infections with additional pathogens could worsen the clinical prognosis, masking the potential beneficial effects of Siglec‐1 truncation in HIV‐1 mono‐infected individuals. In the present study, we explore the concept of antagonistic pleiotropy (Carter & Nguyen, 2011) in co‐infected individuals, where the potential beneficial effect of lacking Siglec‐1 receptor for avoiding HIV‐1 dissemination might be eclipsed by the deleterious effect of this particular null variant through suboptimal immune control of a bacterial co‐infection with *Mycobacterium tuberculosis* (Mtb), the etiological agent for tuberculosis (TB). This study provides a novel molecular basis to explain how the capacity of Siglec‐1 to bind sialylated ligands initiates immunity against Mtb through recognition of extracellular vesicles that transport antigens.

## 2 RESULTS

### 2.1 Increased frequency of the *SIGLEC1* null allele in individuals with extrapulmonary/disseminated TB from an HIV‐1 Cohort

To assess the possible effect of the *SIGLEC1* null variant in HIV‐1 related co‐infections, we searched for the co‐occurrence of infections in the Swiss HIV‐1 Cohort Study (SHCS). We analyzed the clinical records of 3732 participants, in which we had previously identified 85 heterozygous and two homozygous individuals bearing the *SIGLEC1* null variant (Martinez‐Picado et al., 2016). The characteristics of these patients have been described elsewhere (Martinez‐Picado et al., 2016). It is important to highlight that the SHCS follows the classification of the Center for Disease Control and Prevention (CDC) for AIDS‐defining opportunistic infections, and has collected these records since 1988. To evaluate if the *SIGLEC1* null variant was over‐represented in any particular infectious disease category, we used a hypergeometric test. This analysis revealed that among the 708 individuals from the SHCS with reported bacterial, fungal, viral or protozoal co‐infections, the *SIGLEC1* null variant was significantly associated to Mtb infection (*P*‐value 0.011; Table 1).

**TABLE 1:** Siglec‐1 null variant and AIDS‐defining infectious diseases in the HIV‐1 cohort

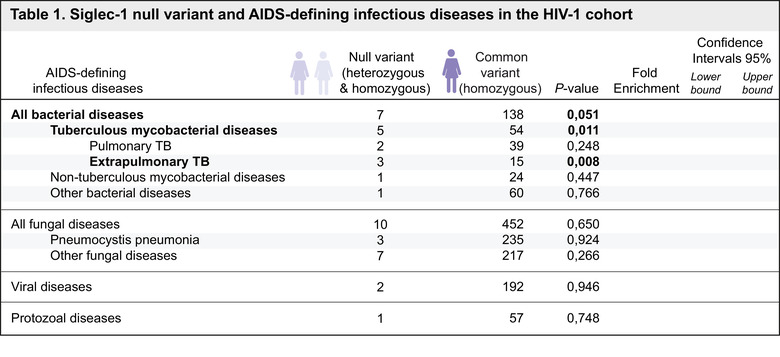

All individuals from the HIV‐1 cohort (n = 3,732) that developed an AIDS‐defining infectious event (n = 708) were analyzed in this table. Categories highlight those infectious diseases in which at least 2 individuals were identified. The association between the infectious diseases detailed in this table and the *SIGLEC1* null variant was assessed with a hypergeometric test. Fold enrichment of the null variant among each of the indicated co‐infections and 95% confidence intervals are also reported. All bacterial infectious diseases include: recurrent bacterial pneumonia, recurrent Salmonella septicaemia, tuberculous mycobacterial diseases, and non‐tuberculous mycobacteria (*M. genavense, M. kansasii, M. avium intracellulare*, and other disseminated mycobacterial diseases). Individuals that developed a tuberculous mycobacterial disease (n = 59) are distinguished by their clinical form of TB. Individuals with concomitant diagnosis of pulmonary and extrapulmonary TB were included in the extrapulmonary TB category. All fungal diseases include: oesophageal and pulmonary candidiasis, disseminated histoplasmosis, *Pneumocystis jiroveci* pneumonia, cryptococcal meningitis and other disseminated cryptococcosis. Viral diseases include: Cytomegalovirus retinitis, other Cytomegalovirus disease, visceral Herpes simplex disease, chronic mucocutaneous Herpes simplex ulceration, Kaposi's sarcoma and progressive multifocal leukoencephalopathy. Protozoal diseases include: isosporiasis, cryptosporidiosis and cerebral toxoplasmosis.

**FIGURE 1:**
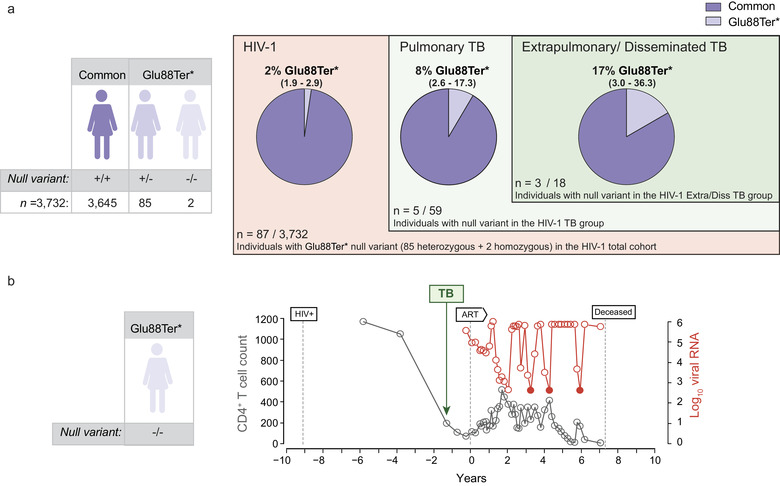
Increased frequency of the *SIGLEC1* null allele in individuals with extrapulmonary/disseminated TB from an HIV‐1 Cohort. a. Representation of the different subgroups analyzed in the HIV cohort. Estimated proportion and 95% confidence interval of individuals harbouring the *SIGLEC1* Glu88Ter* or null allele, including 85 heterozygous and two homozygous individuals. Subjects are grouped by TB diagnosis and by the localization of TB into pulmonary or extrapulmonary/disseminated forms. b. Clinical evolution of a Siglec‐1 null homozygous individual from the HIV‐1 Cohort diagnosed with TB. Dynamics of the CD4^+^ T‐cell count (cells/mm^3^) and plasma viral RNA level (copies/ml) of a Siglec‐1 null homozygous individual from the SHCS cohort, who was diagnosed with pulmonary and extrapulmonary TB. The dates of first HIV‐1 positive report, of TB diagnosis and of antiretroviral treatment (ART) initiation are depicted. Coloured circles indicate values that are below the limit of detection of the viral load. Graph is adapted from Figure 3c of (Martinez‐Picado et al., 2016)

We next classified TB cases in the SHCS cohort by the site of clinical manifestation of the disease into pulmonary or extrapulmonary/disseminated TB forms, and calculated the proportion of individuals bearing the null variant and the 95% confidence interval (Figure 1a). In these subgroups, the association to the presence of the *SIGLEC1* null allele was only significant for the extrapulmonary/disseminated TB category with an over‐representation of 7.15‐fold compared to the total SHCS cohort (*P*‐value 0.008; hypergeometric test; Table 1). In agreement with this association, we observed that one of the homozygous HIV‐1 infected Siglec‐1 null individuals maintained high CD4^+^ T cell counts in the absence of antiretroviral treatment for several years (Figure 1b, grey circles). However, this patient lost immune control and CD4^+^ T cell counts dropped when she was diagnosed with extrapulmonary/disseminated TB along with a pulmonary TB (Figure 1b, green arrow). The identification of an Mtb‐infected *SIGLEC1‐*null homozygote ruled out a requirement for a functional Siglec‐1 for becoming productively infected with Mtb. Yet, the lack of Siglec‐1 in this particular homozygous individual could have had a negative impact upon Mtb infection, complicating HIV‐1 progression (Figure 1b). In the SHCS cohort, the significant association found between the *SIGLEC1* null allele and TB appears to be linked to an extrapulmonary dissemination of Mtb, what prompted us to confirm this particular association in an independent clinical cohort.

### 2.2 An increased frequency of the *SIGLEC1* null allele in individuals with extrapulmonary/disseminated TB is also confirmed in a TB cohort

**FIGURE 2:**
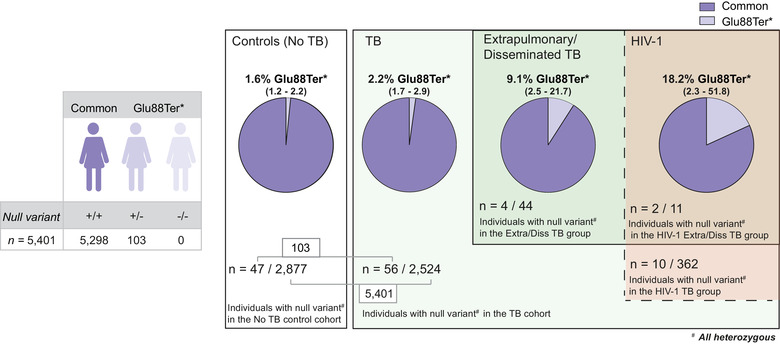
An increased frequency of the *SIGLEC1* null allele in individuals with extrapulmonary/disseminated TB in the HIV‐1 cohort is also confirmed in a TB cohort. Representation of the different subgroups analyzed in the TB cohort. Estimated proportion and 95% confidence interval of individuals harboring the *SIGLEC1* Glu88Ter* or null allele, including 103 heterozygous individuals and no homozygous individuals. In the TB group, individuals are categorized by extrapulmonary/disseminated TB and HIV‐1 status

Next, we determined the proportion of the *SIGLEC1* null allele in an independent TB Russian cohort (Curtis et al., 2015) (Figure 2). DNA samples from 5401 individuals including 2877 control subjects and 2524 individuals with clinical manifestation of TB were genotyped for the *SIGLEC1* null variant previously analyzed in the SHCS HIV‐1 cohort (Figure 2). The proportion of individuals bearing the null variant in this Russian cohort is shown in Figure 2. Although no homozygous individuals were detected in the Russian cohort (Figure 2), the 103 heterozygous individuals bearing the *SIGLEC1* null variant identified should display half the amount of Siglec‐1 expression on their cells (Martinez‐Picado et al., 2016). The demographic characteristics of the TB group and the control group are shown in Supp. Table 1. In this Russian TB cohort, we observed a significant increase of 6‐fold in the frequency of the *SIGLEC1* null allele between the control group and the disseminated TB group (*P*‐value 0.007; Confidence intervals 95% = 1.5‐17.6; Fisher's exact test). We next focused on the TB group, and since it had a subset of HIV‐1 co‐infected individuals (Figure 2), we performed a logistic regression analysis adjusted for HIV‐1 to avoid a possible confounding effect, although no significant association between the *SIGLEC1* null allele and HIV‐1 infection had been previously found (Martinez‐Picado et al., 2016). This analysis revealed only a significant association between the dissemination of Mtb and the *SIGLEC1* null allele (*P*‐value 0.005; odds ratio of 4.55; Confidence intervals 95% = 1.3‐11.9). Overall, data from this independent Russian cohort confirmed the significant association between the disseminated form of TB and the *SIGLEC1* null allele.

### 2.3 Mtb local dissemination in infected *SIGLEC1* knockout mice aggravates pulmonary lesions

**FIGURE 3:**
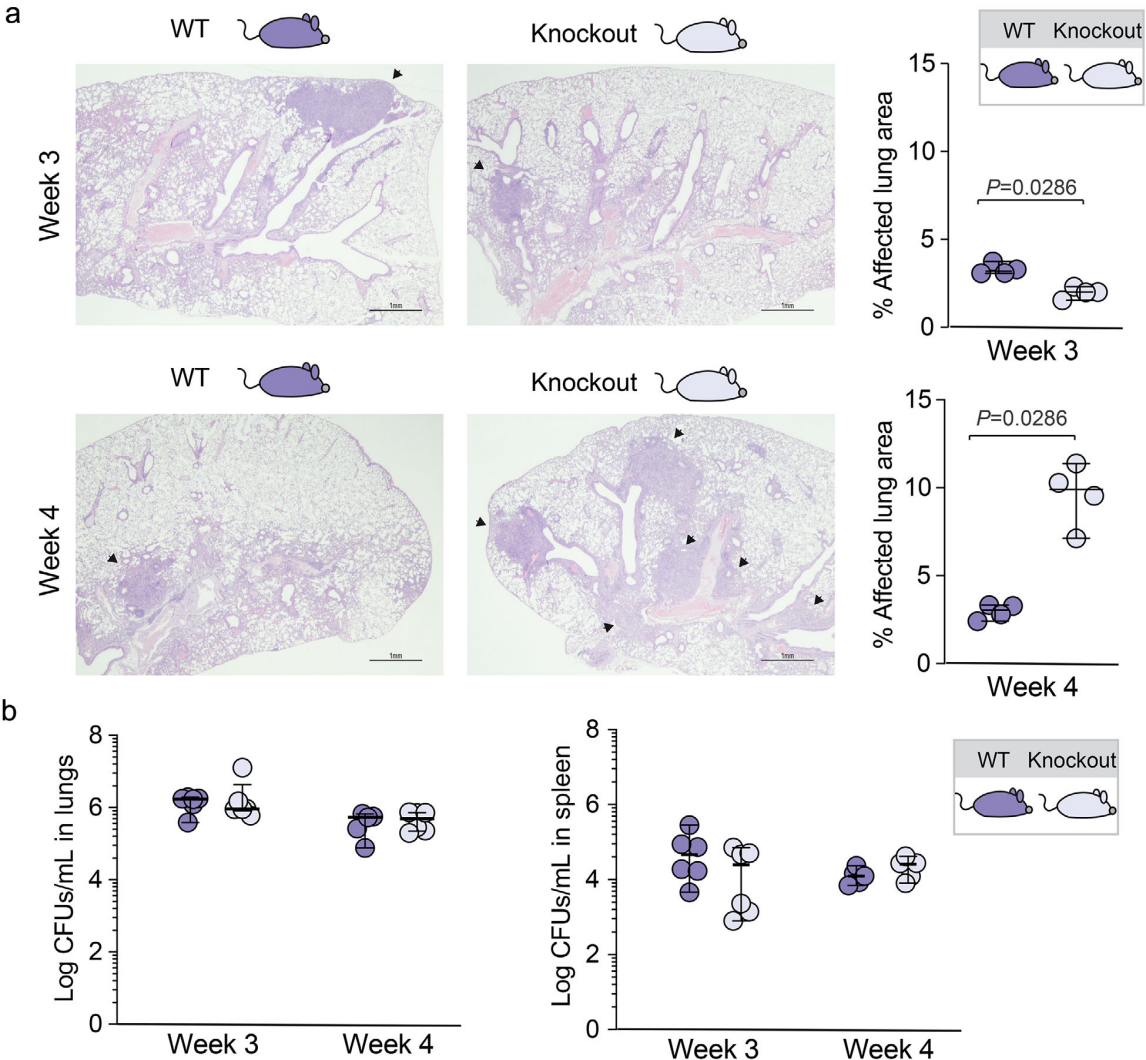
Mtb local dissemination in infected *SIGLEC1* knockout mice aggravates pulmonary lesions. a. Images show representative haematoxylin/eosin staining from lungs of wild type (WT) and *SIGLEC1* knockout C57/BL6 mice challenged via aerosol with Mtb H37Rv at 3‐ or 4‐weeks post‐infection. Arrows mark damaged areas. Graphs show the corresponding quantification of the damaged area as a percentage of the total lung area analyzed. Median values and range are depicted. Statistical differences were assessed with a Mann‐Whitney test. b. Growth of Mtb H37Rv in the lungs and the spleen of wild type and *SIGLEC1* knockout C57/BL6 mice infected with Mtb for 3 or 4 weeks. Results are presented as the medians and range of Log_10_ of bacterial CFUs per ml

To assess the functional association between the *SIGLEC1* null variant and Mtb dissemination, we compared wild type and Siglec‐1 knockout C57BL/6 mice (Oetke et al., 2006) infected with Mtb H37Rv Pasteur strain via aerosol. When the Mtb‐affected areas of the lungs of these mice were compared to the total lung area by histopathologic analysis (Figure 3a, images) Siglec‐1 knockout mice had slightly smaller affected areas at week 3 post‐infection (p.i.), but a much higher percentage of damaged tissue than wild type mice at week 4 p.i. (Figure 3a, graphs, *P*‐value 0.029; Mann‐Whitney test). These results indicate that *SIGLEC1* knockout mice have a delayed generation of pulmonary lesions at week 3 p.i., which are bigger and less structured at week 4 p.i. when compared to wild type mice. Of note, the bacillary load in lungs was similar in both wild type and *SIGLEC1* knockout animals at weeks 3 and 4 p.i. (Figure 3b), indicating that Siglec‐1 is dispensable for becoming infected with Mtb in the mouse model as already observed in human cohorts. Equivalent bacillary load was also detected in spleen, denoting that Mtb similarly reached lymphoid tissues in both mouse strains at weeks 3 and 4 p.i. (Figure 3b). Accordingly, no significant differences between wild type and *SIGLEC1* knockout mice were observed in cytokine production detected in serum or lung homogenates (Supp. Fig. 1 and 2). Results in the mouse model indicate that the absence of Siglec‐1 might unfavourably affect bacterial containment within the lung but without detectable changes in bacterial load or cytokine environment.

**FIGURE 4:**
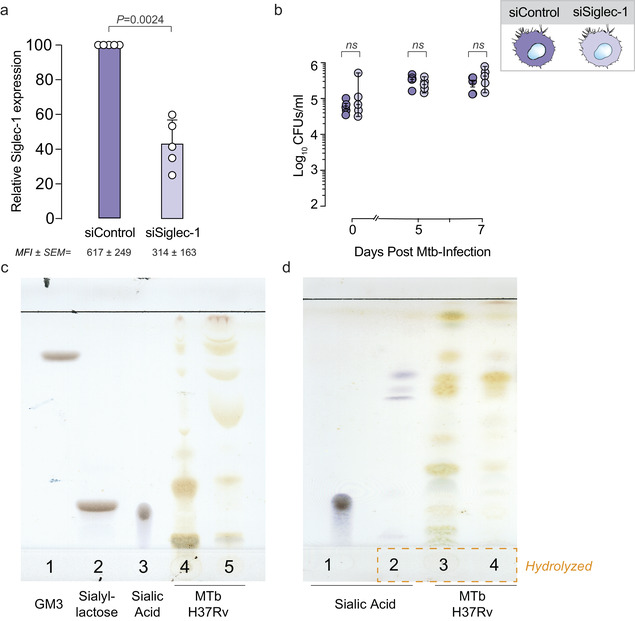
Siglec‐1 is not involved in Mtb capture and clearance. a. Relative Siglec‐1 expression on blood‐derived monocytes transfected with a silencing siRNA control (siControl) or with a Siglec‐1‐targeting siRNA (siSiglec‐1). Siglec‐1 geometric mean fluorescence intensity (MFI) was assessed by flow cytometry and values were normalized to control monocytes (set at 100%). Numbers indicate mean values and standard error of the means (SEM) obtained from five donors tested in triplicates. Statistical differences were assessed with a one sample *t*‐test. b. Kinetic analysis of Mtb replication (CFU/ml) in monocyte‐derived macrophages silenced with a siRNA control (siControl) or with a Siglec‐1‐targeting siRNA (siSiglec‐1). Results are presented as Log_10_ medians of bacterial CFUs and range. Statistical differences were analyzed using linear models of mixed effects. No significant differences were observed between the values of siControl and siSiglec‐1 either looking at different time points or focusing on the slope of growth. c. Thin‐layer chromatography of GM3 standard (lane 1); Sialyllactose standard (lane 2); Sialic acid standard (lane 3); aqueous extracts of Mtb H37Rv (lane 4) and chloroform extracts of Mtb H37Rv (lane 5). Plate was revealed with resorcinol spray that is specific for sialic acid and detected by brown‐violet or blue‐violet colours. D. Thin‐layer chromatography of sialic acid standard (lane 1); hydrolyzed sialic acid standard (lane 2); aqueous extracts of hydrolyzed Mtb H37Rv (lane 4) and chloroform extracts of hydrolyzed Mtb H37Rv (lane 5). Plate was revealed with resorcinol spray that detects sialic acid by brown‐violet or blue‐violet colours

### 2.4 Siglec‐1 is not involved in Mtb capture and clearance

We next tested if this local pulmonary bacterial spread could be related to a lack of direct phagocytic clearance of bacteria via Siglec‐1 receptor activity. Given the reported capacity of Siglec‐1 to uptake sialylated bacteria and restrict pathogen dissemination through clearance (Chang et al., 2014), we evaluated whether Siglec‐1 could directly interact with Mtb to limit local spread and contain the extrapulmonary colonization of bacteria that we found associated to a reduced expression of Siglec‐1 in two independent cohorts. We therefore assessed direct Mtb uptake on Siglec‐1‐expressing human monocyte‐derived macrophages isolated from blood, using RNA interference to reduce Siglec‐1 expression levels. With this strategy, we were able to reduce the cell‐surface receptor expression by 57 ± 14 % (Figure 4a; *P*‐value 0.002; one sample *t*‐test), similar to the cell‐surface expression reported for Siglec‐1 heterozygous individuals bearing the null allele (Martinez‐Picado et al., 2016). These macrophages, along with corresponding non‐targeted RNA controls, were infected with Mtb H37Rv Pasteur strain *in vitro* and the bacillary load was measured over time. We found no significant differences between the two conditions in bacterial uptake at day 0 or over time (Figure 4b), regardless of the RNA interference status. These results further confirmed our previous observations in mice, where no differences in bacillary loads were found (Figure 3b). These findings also suggest that Siglec‐1 is not directly involved in Mtb clearance.

As an alternative approach to asses a putative interaction between Siglec‐1 and Mtb, we next focused on the study of Siglec‐1 interacting ligands on the bacteria. We were not able to find prior reports describing the presence of sialylated molecules on Mtb that could interact with Siglec‐1. Thus, we analyzed their presence in bacterial extracts using thin‐layer chromatography. Compared to the positive detection of sialic acid by blue‐violet spots on several sialylated standards used (Figure 4c, lines 1–3; corresponding to GM3‐sialyllactose containing lipids, sialyllactose, and sialic acid molecules), no positive blue signals were detected in chloroform or aqueous extracts from Mtb H37Rv (Figure 4c, lines 4–5). To further release any possible sialic acid bound to other bacterial components and increase the likelihood of detection, we next performed an acid hydrolysis of Mtb H37Rv extracts. While on the sialic acid standard (Figure 4d, line 1) this hydrolysis yielded blue‐violet spots corresponding to sialylated compounds (Figure 4d, line 2), no spot with comparable chromatographic appearance was detected on hydrolyzed Mtb H37Rv extracts (Figure 4d, lines 3–4). Thus, thin‐layer chromatography analysis did not detect the presence of sialylated ligands associated to Mtb that could directly bind to Siglec‐1. Taken together, our experiments suggest no direct interaction between Siglec‐1 and Mtb in functional cassays and a lack of sialylated ligands on Mtb. These findings indicate that beyond the direct clearance of sialylated pathogens, alternative immune mechanisms against Mtb might be triggered by Siglec‐1 receptor and could be altered on individuals bearing the *SIGLEC1* null variant.

### 2.5 Siglec‐1 on antigen‐presenting cells is required to induce antigen presentation via extracellular vesicle uptake

Siglec‐1 is able to bind and capture sialylated extracellular vesicles *in vitro* (Díaz‐Varela et al., 2018; Izquierdo‐Useros et al., 2012) and *in vivo* (Saunderson et al., 2013). Vesicles secreted by infected cells or antigen‐presenting cells allow for the transfer of processed antigens to dendritic cells, which amplify the initiation of immune responses (Théry et al., 2002, 2009; Yáñez‐Mó et al., 2015). We hypothesized that Siglec‐1 on antigen‐presenting cells could be critical for trapping extracellular vesicles that are involved in the initiation or amplification of the immune response against Mtb (Bhatnagar & Schorey, 2007; Bhatnagar et al., 2007; Cheng & Schorey, 2013, 2019; Singh et al., 2012; Smith et al., 2017).

**FIGURE 5:**
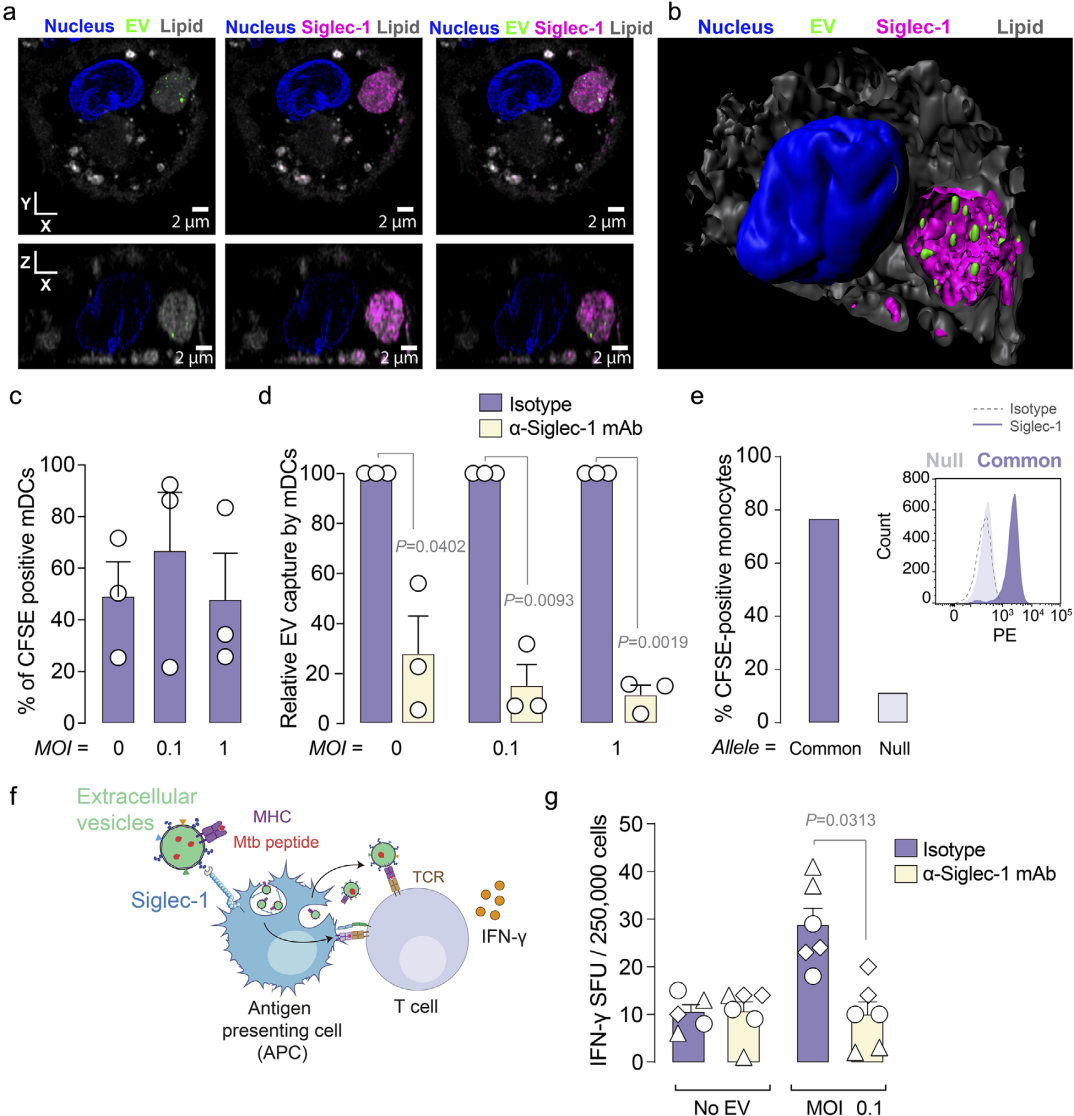
Siglec‐1 on antigen‐presenting cells is required to induce antigen presentation via extracellular vesicle uptake. a. Confocal microscopy analysis of mDCs pulsed with CFSE‐labelled extracellular vesicles (EV; green) purified from Mtb‐infected THP‐1‐derived macrophages. mDCs were stained with an anti‐Siglec‐1 mAb (magenta), with DAPI to detect nuclei (blue) and with lipid dyes to reveal membranes (grey). XY and XZ volume slices of a representative mDC loaded with extracellular vesicles are shown. Scale bar = 2 μm. b. Isosurface rendering of the mDC loaded with extracellular vesicles. c. Flow cytometry analysis of mDC uptake of CFSE‐labelled EV purified from THP‐1‐derived macrophages left uninfected (MOI = 0) or infected with Mtb at a MOI of 0.1 or 1. Mean values and SEM from three independent experiments. d. Relative uptake of CFSE‐labelled EV as in C, where mDCs were preincubated with an anti‐Siglec‐1 mAb or an isotype control. Values are normalized to the level of EV uptake by isotype control‐treated cells (set at 100%). Statistical differences were assessed with a one sample *t*‐test. e. Capture of fluorescent EV by IFN‐α‐activated monocytes isolated from a Siglec‐1 null individual and common allele individual. Histograms show Siglec‐1 expression on these activated monocytes. f. Schematic representation of the co‐culture performed in g. Antigen‐presenting cells such as activated monocytes capture EV from Mtb‐infected THP‐1‐derived macrophages via Siglec‐1. These vesicles can either bear antigens already loaded into MHC molecules, or be re‐processed and presented via MHC‐I or II molecules expressed on the antigen presenting cell capturing these vesicles. Either way, these EVs can lead to the activation of T‐cells. g. IFN‐γ‐producing cells detected in a co‐culture of PBMCs from TB‐infected individuals induced by EVs derived from Mtb‐infected THP‐1‐derived macrophages. Activated monocytes previously pulsed or not with EVs derived from Mtb‐infected cells, in the presence of an anti‐Siglec‐1 mAb or an isotype control, were cultured with autologous CD14^–^ cells. The mean values and standard error of the mean (SEM) of IFN‐γ spot‐forming cells (SFC) per 2.5 × 10^5^ cells are represented. Statistical differences were assessed with a Wilcoxon test. Data from three patients assessed in duplicate are shown

We therefore tested if extracellular vesicles released by Mtb‐infected cells could be captured by one of the most potent antigen‐presenting cells located in the lymphoid tissues, such is the case of human mature dendritic cells (mDCs). Our first approach was to characterize the vesicles derived from Mtb‐infected cells using cryo‐electron microscopy and nanoparticle tracking analysis to define their diameter and size (Supp. Figure 3). Next, we generated the same extracellular vesicles but this time loaded with fluorescence to assess their capture by mDCs using confocal microscopy (Figure 5a‐b). These extracellular vesicles were captured by mDCs and accumulated in sac‐like compartments after 4 h (Figure 5a‐b and Movies 1 and 2). This is in agreement with previously described work on extracellular vesicles isolated from different sources (Díaz‐Varela et al., 2018; Izquierdo‐Useros et al., 2009) and for very distinct sialylated viruses (Akiyama et al., 2014; Erikson et al., 2015; Kijewski et al., 2016; Perez‐Zsolt et al., 2019) that subvert this pathway (Izquierdo‐Useros et al., 2009). The percentage of fluorescent‐positive mDCs was also assessed by flow cytometry and was similar regardless of whether the vesicles were produced by cells infected with Mtb at a high or low multiplicity of infection or by uninfected cells (Figure 5c). Then, mDCs were incubated with fluorescent extracellular vesicles in the presence or absence of a blocking monoclonal antibody (mAb) against Siglec‐1 (Figure 5d). While pre‐treatment with an isotype mAb control had no effect on extracellular vesicle uptake, treatment with an anti‐Siglec‐1 mAb inhibited retention of these vesicle couriers (Figure 5d; *P*‐values on the graph; one sample *t*‐test). When monocytes isolated from homozygous *SIGLEC1* null and common allele individuals were cultured in the presence of IFN‐α to induce Siglec‐1 expression, and then exposed to fluorescent extracellular vesicles, cells from the null individual which naturally lack Siglec‐1 expression (Figure 5e, histograms) did not capture extracellular vesicles (Figure 5e, bars). Therefore, Siglec‐1 is required for extracellular vesicle capture by mDCs and activated monocytes, which are key cells implicated in the initiation of immunity in secondary lymphoid tissues (León et al., 2007; Steinman, 2007; Steinman & Banchereau, 2007).

Finally, we assessed if antigen‐presenting cells that had captured extracellular vesicles through Siglec‐1 could effectively trigger immune responses. We performed a functional assay in which CD14^+^ monocytes from Mtb‐infected individuals, cultured in the presence of IFN‐α to induce Siglec‐1 expression and then pre‐incubated or not with a mAb against Siglec‐1, were exposed to extracellular vesicles from Mtb‐infected cells (Figure 5f). After extensive washing, monocytes were co‐cultured with autologous peripheral blood mononuclear cells (PBMCs) depleted of monocytes, and then we measured IFN‐γ production by ELISpot assay (Figure 5g). We found higher IFN‐γ responses in PBMCs co‐cultured with monocytes that had captured extracellular vesicles through Siglec‐1 as compared to monocytes that had been pre‐incubated with an anti‐Siglec‐1 mAb (Figure 5g; *P*‐value 0.031; Wilcoxon test). Collectively, these results indicate that adaptive responses can be triggered via Siglec‐1 uptake of extracellular vesicles.

## 3 DISCUSSION

Here we investigated the effect of a *SIGLEC1* null variant in humans and found a significant association with an extrapulmonary dissemination of Mtb in two independent clinical cohorts. To further gain insights into this association, we studied a murine C57BL/6 model infected with Mtb, and compared *SIGLEC1* knockout versus wild type mice. Of note, as opposed to other mouse strains, this particular murine model is highly resistant to TB progression once specific immune responses are mounted 4 weeks p.i. in the lungs (Medina, 1998). Yet, and despite the limitation of using a murine model that only reflects the human course of TB infection partially, our results showed that in the absence of Siglec‐1, the affected area of murine pulmonary lesions assessed by histopathology was larger. This could not be explained by the presence of a higher bacillary load, a lack of bacterial migration towards secondary lymphoid tissues such as the spleen, or reflected in a distinctive cytokine production profile. Thus, in a resistant murine model, the *SIGLEC1* knockout has lower capacity to contain the granulomatous infiltration in the lung. Although this did not represent a difference in the bacterial dissemination towards the secondary lymphoid tissues at the early stages analyzed here, we cannot exclude that this local spread may favour later dissemination in chronic phases.

Since Siglec‐1 is a sialic acid binding scavenger receptor implicated in the uptake and clearance of distinct sialylated bacteria like *Campylobacter jejuni*, group B Streptococcus or certain meningococcus (Chang et al., 2014; Heikema et al., 2010; Jones et al., 2003; Klaas et al., 2012;), we tested whether Mtb could be phagocytosed through Siglec‐1. However, we did not detect a direct interaction between Siglec‐1 and Mtb. Hence, neither the local pulmonary Mtb spread in the mice model nor the extrapulmonary dissemination of Mtb in humans could be attributed to the absence of phagocytic Mtb clearance via Siglec‐1 receptor. Nonetheless, Siglec‐1 expressed on antigen‐presenting cells was required to trap extracellular vesicles derived from Mtb‐infected cells. There, we observed that T‐cell immunity was enhanced via extracellular vesicle capture by Siglec‐1, as IFN‐γ responses were reduced in the presence of an anti‐Siglec‐1 mAb. Thus, in *SIGLEC1* homozygotes for the common allele, the initiation of immunity against Mtb may be mounted earlier due to the amplification of responses via antigen exchange through extracellular vesicles. However, in individuals bearing the *SIGLEC1* null allele, the initiation of immunity against Mtb may be delayed, facilitating the extrapulmonary dissemination of Mtb in humans or favouring the local pulmonary spread of Mtb in mice, just before immunity is mounted likely through Siglec‐1 independent pathways. This extracellular vesicle‐exchange mechanism may be especially relevant for infections in which antigen presenting cells are productively infected and bacteria actively inhibit antigen presentation, as is the case of Mtb (Baena & Porcelli, 2009). Indeed, cell‐cooperation between infected migratory DCs and resident lymph node DCs is necessary for optimal activation of naïve T cells during TB infection, and the active transfer of mycobacterial antigens between these cells is required for T‐cell priming (Srivastava & Ernst, 2014).

Prior reports showed that extracellular vesicles derived from Mtb‐infected cells contain antigenic bacterial proteins (Giri et al., 2010), which modulate antigen presentation during Mtb infection (Bhatnagar & Schorey, 2007; Bhatnagar et al., 2007; Cheng & Schorey, 2013,2019; Singh et al., 2012; Smith et al., 2017). This particular immune activity of extracellular vesicles, which are efficiently trapped by Siglec‐1^5,21,20^, provides a molecular basis to explain the genetic association found between the *SIGLEC1* null variant and the Mtb dissemination. Although different *SIGLEC1* polymorphisms have been previously associated with pulmonary TB in a small sized cohort (Souza De Lima et al., 2017), the functional consequences of those particular variants on Siglec‐1 activity remain to be elucidated. Here, however, we provide a plausible molecular model to explain how Siglec‐1 capacity to bind extracellular vesicles can initiate immune responses against TB (Supp. Figure 4), and how its absence impacts Mtb dissemination. Delayed onset of those responses in individuals lacking this particular receptor allows for extrapulmonary dissemination of Mtb in humans or local pulmonary spread of Mtb in resistant mice. While this hypothesis is compatible with all the observations of this study, additional mechanisms may also be at play. Siglec‐1 is an adhesion receptor that favours attachment between immune cells (Crocker et al., 2007), and therefore, within pulmonary lesions, it could be involved in anchoring infected Siglec‐1‐expressing macrophages with other cells or the extracellular matrix of the granuloma. In this case, its total or partial absence could facilitate infected‐cell migration, leading to a more widespread pulmonary damage. Yet, if Siglec‐1 was just acting as a docking molecule, we should have found higher bacillary loads in the spleen of knockout mice at week 3, when immune responses are still being mounted. Future work should dissect the relative contribution of these Siglec‐1 related pathways to the local pulmonary and extrapulmonary dissemination of Mtb. In turn, the functional study of naturally occurring human ‘knockouts’ in infectious contexts may identify novel host factors, such as Siglec‐1, which illuminate previously unknown pathways that modulate complex infections such as TB.

## 4 MATERIALS & METHODS

### 4.1 Ethics statement

The institutional review board on biomedical research from the Germans Trias i Pujol University Hospital (HUGTiP) approved this study. Participants of the Swiss HIV Cohort Study and the Russian Cohort gave written consent to the cohort study and genetic analyses, as approved by the corresponding local Ethics Committees.

### 4.2 Study cohorts

The frequency of the particular single nucleotide null variant of *SIGLEC1* in the general population is described in the genome aggregation database (https://gnomad.broadinstitute.org/variant/20-3687141-C-A). The association of the *SIGLEC1* null variant Glu88Ter with TB was investigated in two different cohorts. First, the Swiss HIV Cohort Study (SHCS; www.shcs.ch), which comprises 3732 participants that are part of a large cohort study prospectively enrolling HIV‐1 infected individuals since 1988 in Switzerland (Schoeni‐Affolter et al., 2010). Demographics, route of transmission, AIDS defining illnesses, co‐morbidities, and behavioural, clinical and laboratory data are systematically and prospectively collected. Plasma and cell samples are stored longitudinally every 6–12 months. To date, more than 20,000 persons have been enrolled. In the SHCS, TB diagnosis was confirmed by culture, or by combination of response to specific treatment and the presence of acid‐fast bacilli in sputum, or by compatible clinical criteria. Selection criteria for pulmonary and extrapulmonary TB are detailed in the SHCS cohort webpage, and are in accordance to the CDC categories. In our study, individuals with concomitant pulmonary and extrapulmonary/disseminated TB were categorized in the extrapulmonary/disseminated TB group.

The second cohort was from Russia, and it includes 2538 TB patients attending civilian TB dispensaries and TB clinics, along with 2877 healthy subjects recruited in the blood transfusion services of Samara (Russia). TB patients were initially diagnosed based on information regarding TB contact, medical history, clinical symptoms (cough, haemoptysis, chest pain, fever, weight loss), presence of acid‐fast bacilli in sputum smear and characteristic symptoms and signs of pulmonary TB on chest X‐rays, as described previously (Casali et al., 2014; Curtis et al., 2015; Szeszko et al., 2007). Diagnosis was confirmed by culture of Mtb from sputum; otherwise patients were excluded. Of note, sputum and chest X‐ray diagnoses limited the capacity to enrol patients presenting only extrapulmonary/disseminated TB, which are underrepresented in this particular cohort. In the Russian cohort, extrapulmonary/disseminated tuberculosis refers to either lymphatic or haematogenous migration of the bacteria to distant pulmonary areas or different tissues. Individuals with concomitant pulmonary and extrapulmonary/disseminated TB were categorized in the extrapulmonary/disseminated TB group. All individuals lacking information regarding the clinical form of TB were excluded from the analyses, so the final TB cohort included only 2524 individuals.

### 4.3 *SIGLEC‐1* genetic analyses

Genotyping for the HIV‐1 Swiss cohort SHCS has been previously described (Martinez‐Picado et al., 2016). For the Russian cohort, genomic DNA was isolated from frozen whole peripheral blood using the Gentra Puregene Blood Isolation kit (Qiagen) according to the manufacturer's protocol. Samples from individuals with TB diagnosis and control subjects were genotyped for the *SIGLEC1* null variant Glu88Ter (SNP rs150358287C > A) in the *SIGLEC1* gene by TaqMan SNP Genotyping Assay (Assay ID: C_167368973_10; Applied Biosystems). Reactions were performed in a StepOnePlus Real‐Time PCR System (Applied Biosystems) and results were analyzed using StepOne Software v2.3 (Applied Biosystems).

### 4.4 Mice and Mtb infection

Six‐week‐old male C57BL/6 Siglec‐1 wild type and knockout mice were shipped from the Division of Cell Signaling and Immunology, School of Life Sciences, University of Dundee, UK. Animal procedures were carried out by the Tuberculosis Experimental Unit and performed according to the protocol DMAH9071, which was reviewed by the Animal Experimentation Ethics Committee of HUGTiP (registered as B9900005) and approved by the *Departament d'Agricultura, Ramaderia, Pesca, Alimentació i Medi Natural* of the Catalan Regional Government, according to current national and European Union legislation regarding the protection of experimental animals. Mice were supervised daily following a strict monitoring protocol in order to ensure animal welfare, and euthanized, if required, with isoflurane (inhalation excess). Twelve wild type and Siglec‐1 knockout C57BL/6 mice were aerosol challenged with the Mtb H37Rv Pasteur strain using an airborne infection apparatus (Glas‐col Inc.) delivering around 50 colony forming units (CFU). Three and four weeks after challenge, six wild type and knockout mice were euthanized.

### 4.5 Murine histopathological analysis

Right upper lung lobe samples were fixed in formaldehyde (Biopsafe), embedded in paraffin and cut in 5‐μm sections that were stained with haematoxylin‐eosin for microscopic observation (Nikon Instruments Inc.). Four distinct sections of each tissue block were used to determine the damaged area as percentage of the total lung area using the NISElements D version 3.0x software package (Nikon Instruments Inc.).

### 4.6 Murine bacillary load measurement

Lung and spleen samples collected from each animal were mechanically homogenized and plated using serial dilutions on nutrient Middlebrook 7H11 agar plates (BD Diagnostics). Visible CFU were counted after incubation for 28 days at 37°C.

### 4.7 Murine cytokine profiling

Frozen serum samples and snap‐frozen lung homogenates from the right lower and middle lung lobes were assessed. Lung samples were thawed, weighted and homogenized on ice with lysis buffer containing 0.05% sodium azide, 0.5% Triton X‐100, 1:500 Protease inhibitor cocktail (all from Sigma‐Aldrich) in sterile PBS, using 1 ml per 100 mg of tissue. Homogenates were incubated for 1 h at 4°C and centrifuged at 3000 × *g* for 10 min. Supernatants were collected and stored at ‐80°C until use. Cytokines were measured by Luminex xMAP technology and analyzed with xPONENT 3.1 software (Luminex Corporation). IFN‐γ, TNF‐α, IL‐6, LIX and IL‐17 were measured in serum samples, using the MCYTOMAG‐70K kit. In lung homogenates, IFN‐γ, TNF‐α, IL‐4, IL‐6, LIX, IL‐1β, IL‐10, IL‐12, IP‐10, KC, MCP‐1 and VEGF were analyzed with the MCYTOMAG‐70K kit, and TGFβ with the TGFBMAG‐64K kit (EMD Millipore Corporation) following the manufacturer's instructions.

### 4.8 Siglec‐1 siRNA silencing in monocyte‐derived macrophages, Mtb infection and CFU enumeration

PBMCs were obtained from HIV‐1 seronegative donors by Ficoll‐Hypaque density gradient centrifugation, and monocyte populations were isolated with CD14^+^ magnetic beads (Miltenyi Biotec). Silencing in blood‐derived monocytes was performed using reverse transfection protocol as previously described (Troegeler et al., 2014). Monocytes were transfected with 200 nM of ON‐TARGETplus SMARTpool siRNA targeting Siglec‐1 or non‐targeting siRNA control (Horizon Discovery) using HiPerfect transfection system (Qiagen). Cells were left to adhere to glass coverslips (Dominique Dutscher) in 24‐well plates at a density of 0.5 × 10^6^ for 4 h prior to the addition of 0.5 ml of RPMI 1640 (Gibco) supplemented with 10% FBS and 20 ng/ml of human M‐CSF (Peprotech). Culture media was renewed every 3 days for 7 days.

Monocyte‐derived macrophages were then infected with 10^5^ Mtb H37Rv‐DsRed per well (MOI = 0.2) for 4 h at 37°C, washed with PBS to remove extracellular Mtb and replaced with fresh media. At day 0, 5 or 7 post‐infection, cells were washed with PBS and lysed with 0.01% Triton X100 (Sigma‐Aldrich), serially diluted in PBS and plated onto 7H11‐ OADC agar medium (Difco) to assess the bacterial intracellular growth. CFU were determined at days 14 and 21 post‐plating. Of note, a significant increase in bacterial growth over time was observed for all cellular types tested (*P* < 0.0001; likelihood ratio test for time effect in a fitted linear mixed‐effects model). At day 7, part of the cells was assessed for Siglec‐1 expression with an APC‐anti‐Siglec‐1 mAb (BioLegend) or the corresponding isotype control by flow cytometry using BD LSRFortessa flow cytometer (BD Biosciences, TRI Genotoul platform) and the associated BD FACSDiva software. Data were then analyzed using the FlowJo_V10 software (FlowJo, LLC).

### 4.9 Detection of sialylated residues on Mtb cell lysates

Mtb H37Rv Pasteur strain was cultured on Middlebrook 7H11 agar plates (BD Diagnostics) at 37°C. After 3 weeks, mycobacterial cells were harvested and extracted with chloroform, methanol and mixtures of chloroform‐methanol. The extracts were pooled, dried and partitioned with chloroform/methanol/water (8:4:2 v/v). Aqueous phase and chloroform phase were separated and evaporated to dryness. Presence of free GM3, sialyllactose and sialic acid in these extracts was analyzed by thin layer chromatography developed with chloroform/methanol/water (with 0.2% of CaCl_2_) (5:4:1 v/v). Thin layer chromatography plates were revealed with resorcinol, a reagent that specifically stains sialic acid or sialic acid‐derivatives with a distinctive brown‐violet or blue‐violet colour (Waters et al., 1992). Presence of total sialic acid in Mtb H37Rv chloroform and aqueous extracts was determined by acid hydrolysis with 1N HCl in methanol at 80°C for 2 h and thin layer chromatography analysis as explained above. Purified bovine milk ganglioside GM3, sialyllactose and sialic acid working standards (0.025 mg) were obtained from Sigma‐Aldrich.

### 4.10 Mtb infection of THP‐1‐derived human macrophages

THP‐1 were cultured in RPMI with L‐Glutamine containing 10% FBS, 100 U/ml penicillin and 100 μg/ml streptomycin and 100 ng/ml PMA (Sigma‐Aldrich) for 48 h and a 24 h resting period in PMA‐free fresh culture medium to induce macrophage differentiation. Then, 2.2 × 10^6^ THP‐1‐derived macrophages were infected with Mtb H37Rv Pasteur strain at a MOI of 0.1 or 1 for 4 h or left uninfected. After extensive washing, macrophages were cultured for 72 h with extracellular vesicle‐depleted culture medium (Monguió‐Tortajada et al., 2017). Briefly, 20% FBS complete medium was ultra‐centrifuged in polyallomer ultracentrifugation tubes (Thermo Fisher Scientific) at 100,000 × g for > 16 h (TH641 rotor, adjusted k‐Factor = 240.82, Sorvall WX Ultra 100 Series ultracentrifuge, Thermo Fisher Scientific). The supernatant was collected and filtered through a 0.22 μm filter (Sarstedt) to sterilize the medium, which was finally diluted with RPMI medium (1:1) for cell culture.

### 4.11 Extracellular vesicle isolation and purification

All relevant data have been submitted to the EV‐TRACK knowledgebase (EV‐TRACK ID: EV190053) (Van Deun et al., 2017). Extracellular vesicles were isolated from supernatants collected from mock or Mtb‐infected THP‐1‐derived macrophages after 72 h of culture. Supernatants were centrifuged at 400 × *g* for 5 min and at 2000 × *g* for 10 min to exclude cells and cell debris, respectively. Debris‐cleared conditioned medium was then concentrated by 100 kDa ultrafiltration using regenerated cellulose Amicon Ultra (Millipore) at 2000 × *g* for 35 min, obtaining typically 250 μl concentrated conditioned medium. For tracking purposes, extracellular vesicles were stained with 5,6‐Carboxyfluorescein Diacetate Succinimidyl Ester (CFSE; Invitrogen) by incubating the concentrated conditioned medium with 100 μM CFSE for 2 h at 37°C (Morales‐Kastresana et al., 2017). CFSE positive or unstained extracellular vesicles were then isolated from the concentrated conditioned medium (and washed away from free CFSE dye) by size‐exclusion chromatography (SEC) using a modification of a previous method (Monguió‐Tortajada et al., 2017). Briefly, 12 ml of Sepharose CL‐2B (Sigma‐Aldrich) were extensively washed with PBS (Oxoid) and packed in a Puriflash dry load empty 12G flash column (Interchim‐Cromlab). Concentrated conditioned medium was loaded into the SEC, and 500 μl fractions (up to 35) were collected immediately after eluting with PBS. Protein elution was checked by reading absorbance at 280 nm of each fraction using Nanodrop (Thermo Scientific). The presence of extracellular vesicles in the SEC fractions was determined according to their content in tetraspanins by bead‐based flow cytometry, as previously described (Monguió‐Tortajada et al., 2019). Briefly, extracellular vesicles were coupled to 4‐μm aldehyde/sulphate‐latex microspheres (Invitrogen) for 15 min, blocked overnight with BCB buffer (PBS/0.1% BSA/0.01% NaN_3_; all from Sigma‐Aldrich) and spun down at 2000 × *g* for 10 min. Extracellular vesicles‐coupled beads were then labelled with the primary antibodies anti‐CD9 (Clone VJ1/20) and anti‐CD63 (Clone TEA3/18) at 1:100 dilution (kindly provided by Dr. María Yáñez‐Mó from UAM; CBM‐SO, IIS‐IP and Dr. Francisco Sánchez‐Madrid from Hospital Universitario de la Princesa, IIS‐IP, UAM, CNIC) and secondary antibodies Cy5‐conjugated Donkey anti‐Mouse or A647‐conjugated Goat F(ab')2 Anti‐Mouse IgG (Jackson ImmunoResearch), performed at RT for 30 min under mild shaking, washed after each step with BCB buffer and centrifuged at 2000 × *g* for 10 min. Data were acquired in a FACSLyric flow cytometer (BD) and analyzed by FlowJo v.10.2 software (BD). Extracellular vesicles‐containing fractions were pooled together and adjusted to the desired volume with PBS using 100 kDa‐ultrafiltration 2 ml‐Amicon Ultra (Millipore). Extracellular vesicles were kept at 4°C and used within 24 h for the in vitro experiments or frozen (‐1°C/min) at ‐80°C.

### 4.12 Size and morphological analysis of extracellular vesicles

Extracellular vesicles were examined by cryo‐electron microscopy at the Electron Microscopy Service of the Autonomous University of Barcelona. Vitrified specimens were prepared by placing 3 μl of a sample on a Quantifoil 1.2/1.3 TEM grid, blotted to a thin film and plunged into liquid ethane‐N2(l) in a EM CPC cryoworkstation (Leica). The grids were transferred to a 626 Gatan cryoholder and maintained at ‐179°C. Samples were analyzed with a Jeol JEM 2011 transmission electron microscope operating at an accelerating voltage of 200 kV. Images were recorded on a Gatan Ultrascan 2000 cooled charge‐coupled device (CCD) camera with the corresponding Digital Micrograph software package.

Size distribution of particles of EV preparations was determined by nanoparticle tracking analysis in a NanoSight LM10‐12 (Malvern Instruments Ltd), equipped with a 638 nm laser and CCD camera model F‐033. Data were analyzed with software version build 3.1.46, with detection threshold set to 5, and blur, Min track Length and Max Jump Distance set to auto. Samples were diluted in filtered PBS to remove particles in suspension and reach optimal concentration for instrument linearity: 20–120 particles/frame as advised by the manufacturer. Readings were taken on triplicates of 60 s at 30 frames/s, at a camera level set to 16 and with manual monitoring of temperature.

### 4.13 Uptake assays of fluorescent extracellular vesicles

DCs were obtained by culturing monocytes obtained with CD14^+^ magnetic beads (Miltenyi Biotec) in the presence of 1,000 IU/ml GM‐CSF and IL‐4 (both from R&D) for 7 days and replacing media and cytokines every 2 days. At day 5, DCs were matured with 100 ng/ml lipopolysaccharide (*Escherichia coli* O111:B4; Sigma‐Aldrich) to induce Siglec‐1 expression. Uptake experiments with CFSE labelled‐extracellular vesicles were performed by pulsing 2.5 × 10^5^ mDCs for 4 h at 37°C.

For immunofluorescence staining, cells were suspended in 500 μl L‐15 medium, incubated with 100 nM Abberior STAR RED ‐1,2‐dihexadecanoyl‐sn‐glycero‐3‐phosphoethanolamine (DPPE, Abberior GmbH) and washed 3 times with L‐15. Cells were adhered to poly‐L coated coverslips at 37°C and fixed in 3% PFA plus PBS for 15 min. Fixed samples were permeabilized and blocked using 0.1% saponin plus 0.5% BSA and 100 μg/ml human serum IgGs (Privigen, Behring CSL). Cells were immunostained with anti‐Siglec‐1 6H9 mAb (Perez‐Zsolt et al., 2019) directly coupled to Abberior STAR 580 dye (Abberior GmbH). Following immunostaining samples were briefly incubated with NucBlue Live Hoechst 33342 stain (ThermoFisher Scientific) and post‐fixed using 3% PFA. Samples were overlaid with SlowFade Diamond mounting medium (ThermoFisher Scientific) and imaged using confocal microscopy. Confocal microscopy analysis was performed using Zeiss LSM 780 confocal microscope (Jena) equipped with a 63×/1.4 NA oil immersion objective. Image Z‐stacks for each channel were acquired sequentially with the following parameters: pinhole size: 1 Airy, XY pixel size: 50 nm, Z pixel size: 250 nm. Following acquisition images were deconvoluted using Huygens Professional software and theoretical PSF parameters corresponding to the system's effective observation spot or point‐spread‐function. All subsequent image manipulation steps were performed using Fiji (ImageJ distribution) software with the exception of the isosurface generation, which was performed using Imaris.

For flow cytometry analyses, mDCs were left untreated or pre‐incubated for 15 min at RT with 10 μg/ml of anti‐Siglec‐1 mAbs (7‐239 or 7D2; both from Abcam) or IgG1 isotype control (BD Biosciences) before pulse with CFSE labelled‐extracellular vesicles. After extensive washing, mDCs cells were acquired with FACS Calibur (BD Biosciences), and the percentage of positive cells was determined using FlowJo v.10.6 software. Forward and side‐scatter light gating were used to exclude dead cells and debris from all analyses. Monocytes were also isolated with CD14^+^ beads from frozen PBMCs obtained from a homozygous HIV‐1 null individual and common allele HIV‐1 individual with confirmed genotype for the *SIGLEC1* null variant. Monocytes were activated with 1000 U/ml of Interferon‐2α (Sigma‐Aldrich) for 24 h to induce Siglec‐1 expression. 2 × 10^5^ monocytes were pulsed with CFSE labelled‐extracellular vesicles for 4 h at 37°C to assess uptake as described for mDCs. Siglec‐1 expression on these cells was assessed by flow cytometer, blocking cells with 1 mg/ml of hIgGs and staining them with α‐Siglec‐1‐PE mAb 7–239 or matched isotype‐PE control (BioLegend) at 4°C for 30 min. Samples were analyzed with FACSCanto II (BD Biosciences) using FlowJo v.10.6 software.

### 4.14 IFN‐γ ELISpot assay

PBMCs from three HIV‐1 positive individuals with TB were thawed and monocytes were isolated and activated with Interferon‐2α as previously described. The CD14^–^ fraction from the same individual was cultured for 24 h with 20 U/ml IL‐2 (Novartis). Then, monocytes were pre‐incubated for 15 min at RT with 10 μg/ml of an anti‐Siglec‐1 mAb (7D2; Abcam), IgG1 isotype control (BD Biosciences) or left untreated. Monocytes were pulsed for 4 h at 37°C with CFSE labelled‐extracellular vesicles isolated from THP‐1‐derived macrophages infected with Mtb as previously described. After extensive washes, extracellular vesicle capture was confirmed in a FACS Calibur (BD Biosciences).

After extracellular vesicle exposure, 2.5 × 10^4^ monocytes were cultured with 2.5 × 10^5^ autologous CD14^–^ cells per well in duplicate for 48 h to assess IFNγ production using the Human IFNγ ELISpot^PLUS^ kit (Mabtech) following manufacturers’ instructions. Unstimulated cells were used as negative control. PBMCs stimulated with phytohemagglutinin (Sigma‐Aldrich) at 15 μg/ml were used as positive control. Of note, the capacity of CD14^–^ cells to produce IFNγ against Mtb peptides was confirmed by detection of spot forming cells after the addition of 10 μg/ml of purified protein derivative or PPD (AJVacines). The number of spots (representing individual IFNγ producing cells) were counted using an automated ELISPOT reader system (ImmunoSpot S6 Versa; CTL), which was manually validated.

### 4.15 Statistical analyses

The association between the *SIGLEC1* null allele and the TB phenotypes was assessed by an enrichment analysis using a Chi‐squared test, a hypergeometric test, a proportion test or a logistic regression, according to the design of the cohorts. Mean or median changes were analyzed using a paired *t*‐test or a Mann–Whitney U‐test, respectively. Mean changes from 100% of data normalized to percentages were assessed with a one‐sample *t*‐test. Longitudinal values of bacterial replication were analyzed using linear models of mixed effects. Test results were considered significant at *P* < 0.05. All analyses and figures were generated with R or GraphPad Prism v.8 software.

## FINANCIAL SUPPORT

Javier Martinez‐Picado and Nuria Izquierdo‐Useros are supported by the Spanish Secretariat of State of Research, Development and Innovation (SEIDI) through grant SAF2016‐80033‐R. Javier Martinez‐Picado is supported by PID2019‐109870RB‐I00. This research was sponsored in part by Grifols and by CERCA Programme/Generalitat de Catalunya 2017 SGR 252. The genetic analyses were realized within the framework of the Swiss HIV Cohort Study (SHCS Project number 747), which is supported by the Swiss National Science Foundation (Grant Number 177499) and by the SHCS research foundation. Susana Benet is supported by the Rio Hortega programme funded by the Spanish Health Institute Carlos III (No. CM17/00242). Cristina Gálvez is supported by the PhD fellowship of the Spanish Ministry of Education, Culture and Sport (FPU15/03698). Francis Drobniewski is supported by the Imperial Biomedical Research Center and by Horizon 2020 grant No. 825673 CARE: Common Action Against HIV/TB/HCV Across the Regions of Europe. Irina Kontsevaya is also supported by Horizon 2020 CARE grant No. 825673, grant No. 733079 (AnTBiotic: progressing TB drug candidates to clinical proof of concept) and grant No. RIA2017T‐2030 (CLICK‐TB: Novel Clinical Candidates to Kill TB). Jakub Chojnacki is supported by European Union's Horizon 2020 research and innovation programme under the Marie Skłodowska‐Curie grant agreement No. 793830. Lilibeth Arias is supported by the European Commission Horizon 2020 research and innovation program under grant agreement TBVAC2020 No. 643381. Paula Cardona and Cristina Vilaplana are supported by the Plan Nacional I + D + I co‐financed by ISCIII‐Subdirección General de Evaluación and Fondo‐EU de Desarrollo Regional (FEDER) through IFI14/00015 and CPII18/00031. Lilibeth Arias, Paula Cardona, Pere‐Joan Cardona and Cristina Vilaplana are supported by the Catalan Agency for Management of University and Research Grants (AGAUR 2017 SGR500). Geanncarlo Lugo‐Villarino and Christel Vérollet are supported by the ANRS 2020–1 grant. Marta Monguió‐Tortajadaand Francesc E. Borràs are founded by SGR programmes (2017‐SGR‐301 REMAR Group, and 2017‐SGR‐483 ICREC Group) from the Generalitat de Catalunya. Francesc E. Borràs is a researcher from Fundació Institut de Recerca en Ciències de la Salut Germans Trias i Pujol, supported by the Health Department of the Catalan Government (Generalitat de Catalunya). Marina Luquin and Esther Julián are supported by AGAUR grant (2017 SGR‐229). Paul R. Crocker was supported by the Wellcome Trust grant 103744/Z/14/Z.

## CONFLICT OF INTEREST

The authors declare that no competing financial interests exist.

## AUTHOR CONTRIBUTION

Conceived and designed experiments: Susana Benet, Cristina Vilaplana, Nuria Izquierdo‐Useros, Javier Martinez‐Picado. Analyzed and interpreted clinical/molecular data: Susana Benet, Hansjakob Furrer, Hansjakob Furrer, Huldrych F. Günthard, Jacques Fellay, Paul J. McLaren, Amalio Telenti, Cristina Gálvez, Irina Kontsevaya, Francis Drobniewski, Victor Urrea, Judith Dalmau, Bonaventura Clotet, Cristina Vilaplana, Nuria Izquierdo‐Useros, Javier Martinez‐Picado. Performed and analyzed murine experiments: Susana Benet, Itziar Erkizia, Ruo‐Yan Ong, Paul R. Crocker, Gustavo Tapia, Lilibeth Arias, Paula Cardona, Pere‐Joan Cardona, Cristina Vilaplana, Nuria Izquierdo‐Useros, Javier Martinez‐Picado. Performed and analyzed thin‐layer chromatography experiments: Susana Benet, Marina Luquin, Esther Julián, Cristina Vilaplana, Nuria Izquierdo‐Useros, Javier Martinez‐Picado. Performed and analyzed gene silencing experiments in human leukocytes: Susana Benet, Victor Urrea, Maeva Dupont, Geanncarlo Lugo‐Villarino, Christel Vérollet, Cristina Vilaplana, Nuria Izquierdo‐Useros, Javier Martinez‐Picado. Performed and analyzed extracellular vesicle experiments: Susana Benet, Marta Monguió‐Tortajada, Lilibeth Arias, Itziar Erkizia, Jakub Chojnacki, Francesc E. Borràs, Cristina Vilaplana, Nuria Izquierdo‐Useros, Javier Martinez‐Picado. Wrote the paper: Susana Benet, Nuria Izquierdo‐Useros. Performed critical revision: Cristina Vilaplana, Nuria Izquierdo‐Useros, Javier Martinez‐Picado. All authors reviewed and approved the final draft of the paper.

## Supporting information


**Supplementary Figure 1. Comparative cytokine profile measured in serum samples from wild type and Siglec‐1 knockout mice**. Results are expressed as mean and SEM in pg per ml of serum. Statistical differences were assessed with a Mann‐Whitney test.

**Supplementary Figure 2. Comparative cytokine profile measured in lung homogenates from wild type and Siglec‐1 knockout mice**. Results are expressed as mean and SEM in pg per ml of lysate homogenate. Statistical differences were assessed with a Mann‐Whitney test.

**Supplementary Figure 3. Size and morphological analysis confirms the presence of extracellular vesicles. A**. Cryogenic electron microscopy (cryo‐EM) analysis of extracellular vesicles purified from THP‐1‐derived macrophages infected with Mtb at a MOI of 0.1 shows round vesicles with a distinctive membrane and a diameter and size concurring to extracellular vesicles, as quantified in the following panels**. B**. Histogram depicting the distribution of the diameter of the extracellular vesicles according to cryo‐EM. **C**. Histogram showing the size and concentration of extracellular vesicles according to nanoparticle tracking analysis.

**Supplementary Figure 4. Hypothetical model explaining the early induction of immunity against Mtb in the presence or in the absence of Siglec‐1**. **A**. Susceptibility to Mtb infection is similar in both cases, as human and mice are equally infected in the absence of Siglec‐1. Moreover, Mtb replication rates are also equivalent in murine models and human cells with varying Siglec‐1 expression levels. **B**. Once antigen‐presenting cells are infected, these cells migrate to secondary lymphoid tissues, where they are not competent for direct antigen presentation as they are productively infected with Mtb, but can transfer antigens to competent uninfected antigen presenting cells through extracellular vesicle release, which are captured via Siglec‐1. **C**. Antigen uptake by Siglec‐1 amplifies the initiation of T cell responses. **D**. These T‐cell responses are mounted faster and contain the pulmonary damage. **E**. In sharp contrast, the lack of Siglec‐1 compromises antigen exchange via extracellular vesicles. **F**. Thus, in the absence of Siglec‐1, T‐cell responses are mounted later and are not able to control the early pulmonary damage leading to bacterial dissemination.

Supplementary information

**Movie 1**. Isosurface rendering animation of a mDC loaded with extracellular vesicles.

**Movie 2**. Isosurface rendering animation of the sac‐like compartment of the mDC from Movie 1, where Siglec‐1 (magenta) is in the same sac‐like structure where extracellular vesicles (green) are retained.

## Data Availability

The data that support the findings of this study are available from the corresponding authors upon reasonable request.
