# Supplementary figures and images for: Dissemination of Mycobacterium tuberculosis is associated to a SIGLEC1 null variant that limits antigen exchange via trafficking extracellular vesicles
Source: J Extracell Vesicles. 2021 Jan 14;10(3):e12046. doi: 10.1002/jev2.12046 (PMC7807485; doi:10.1002/jev2.12046)

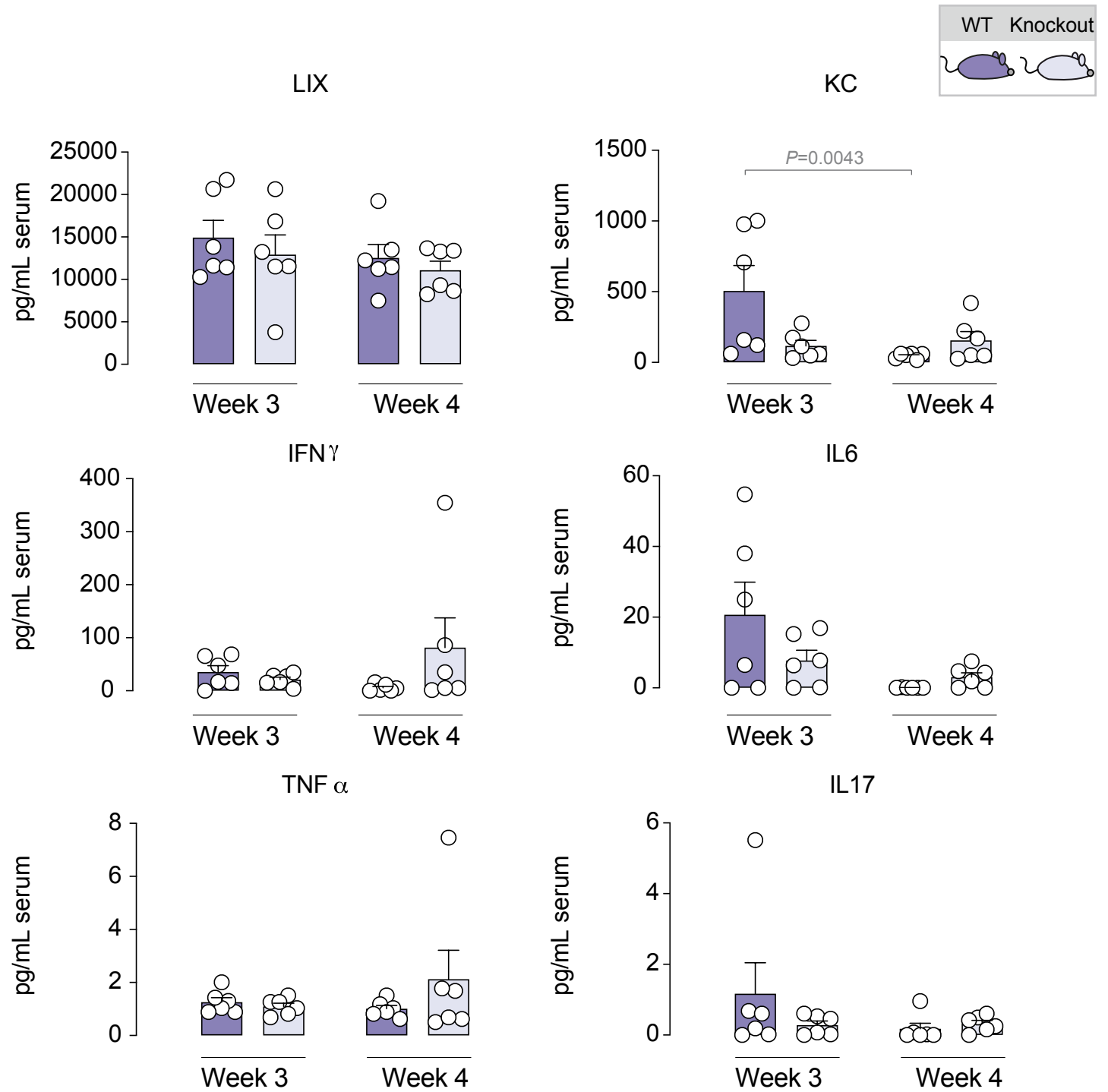

Supplementary Figure 1

Supplement: Supplementary file 1 — Supplementary Figure 1. Comparative cytokine profile measured in serum samples from wild type and Siglec‐1 knockout mice. Results are expressed as mean and SEM in pg per ml of serum. Statistical differences were assessed with a Mann‐Whitney test. [file JEV2-10-e12046-s001.pdf]

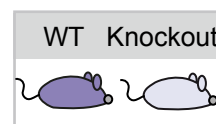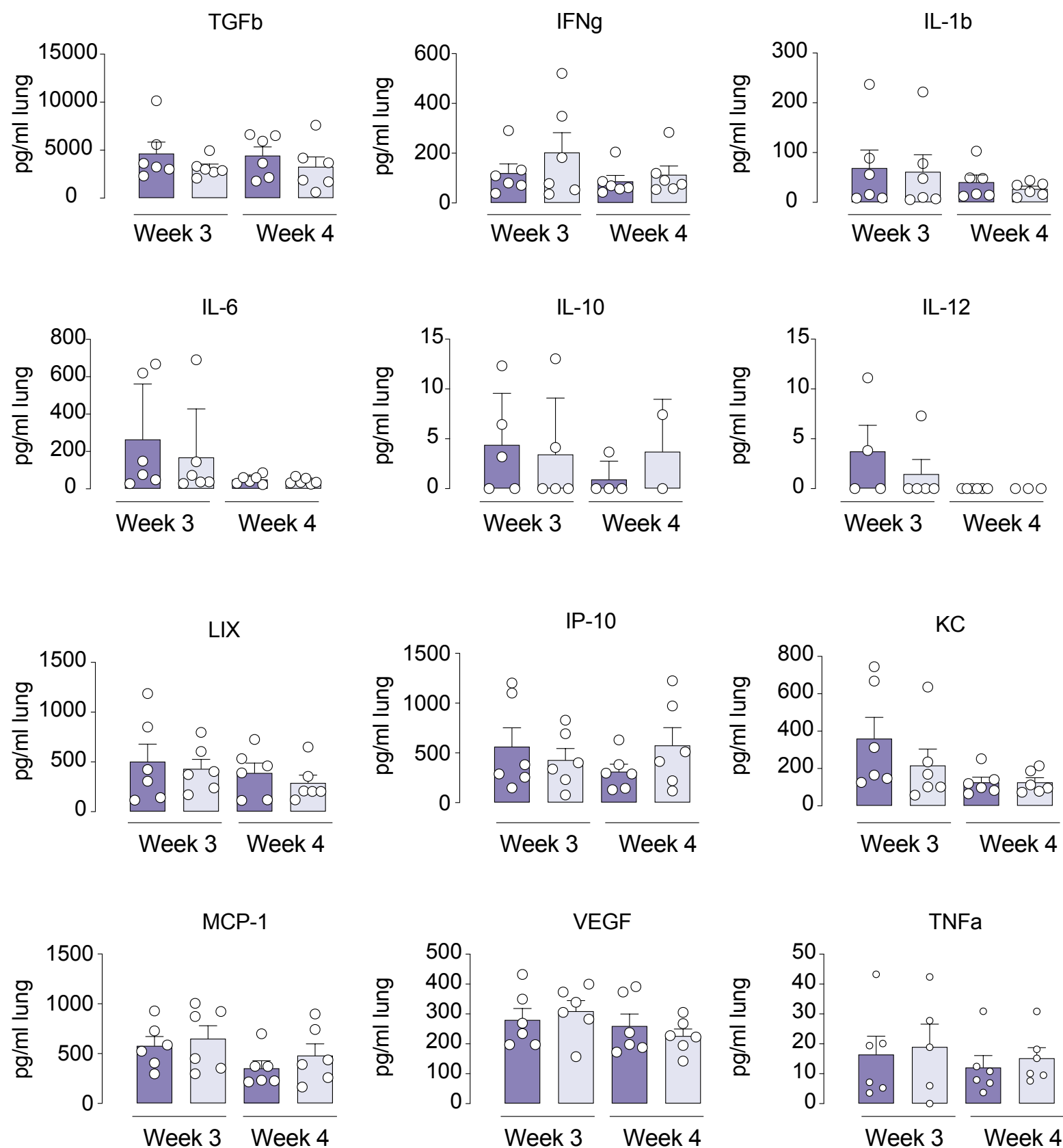

Supplementary Figure 2

Supplement: Supplementary file 2 — Supplementary Figure 2. Comparative cytokine profile measured in lung homogenates from wild type and Siglec‐1 knockout mice. Results are expressed as mean and SEM in pg per ml of lysate homogenate. Statistical differences were assessed with a Mann‐Whitney test. [file JEV2-10-e12046-s002.pdf]

A

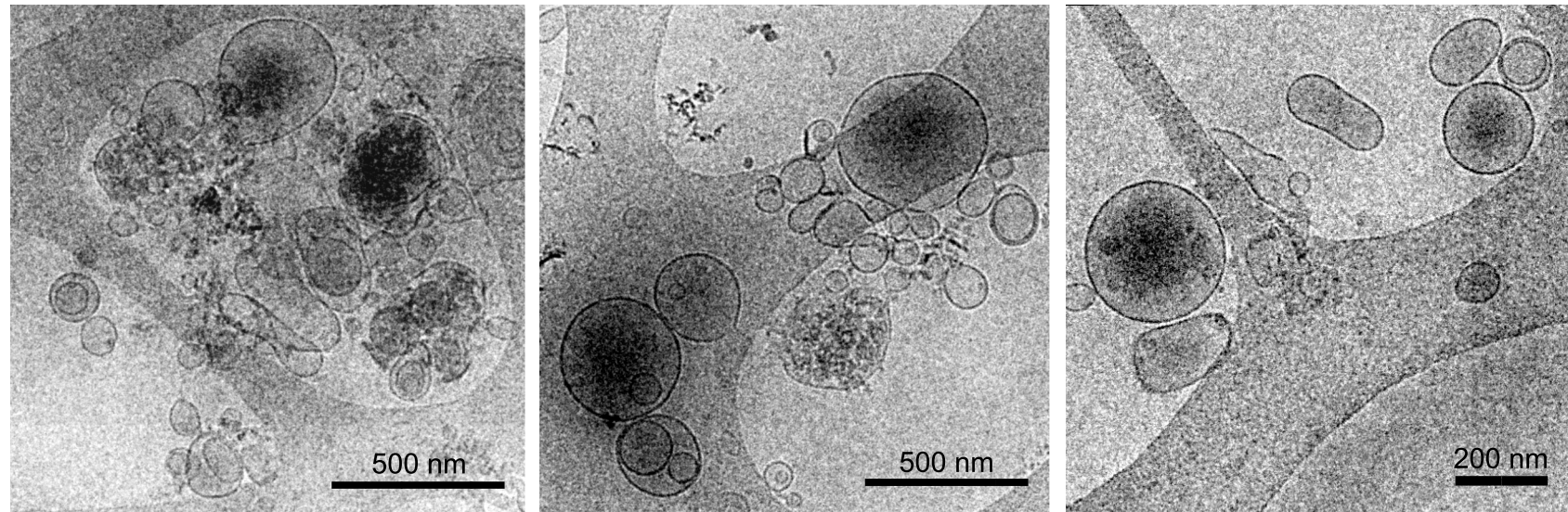

B

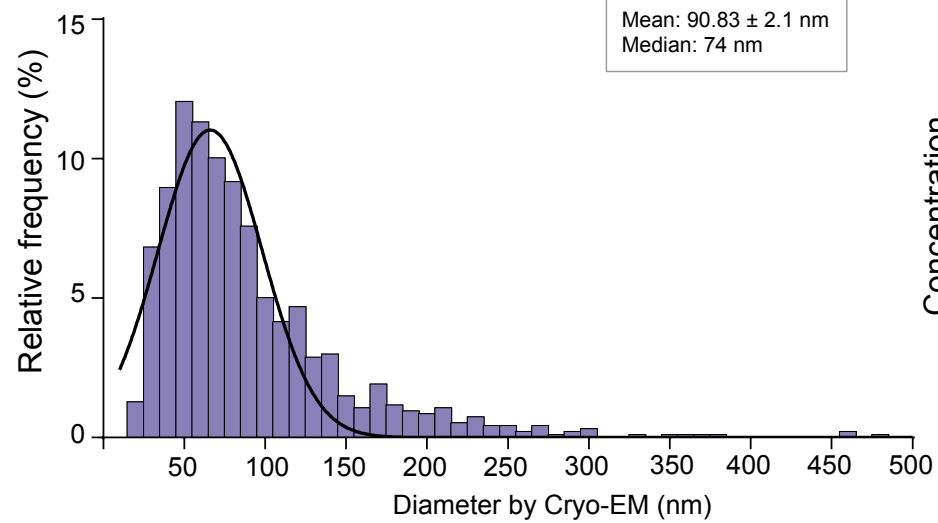

C

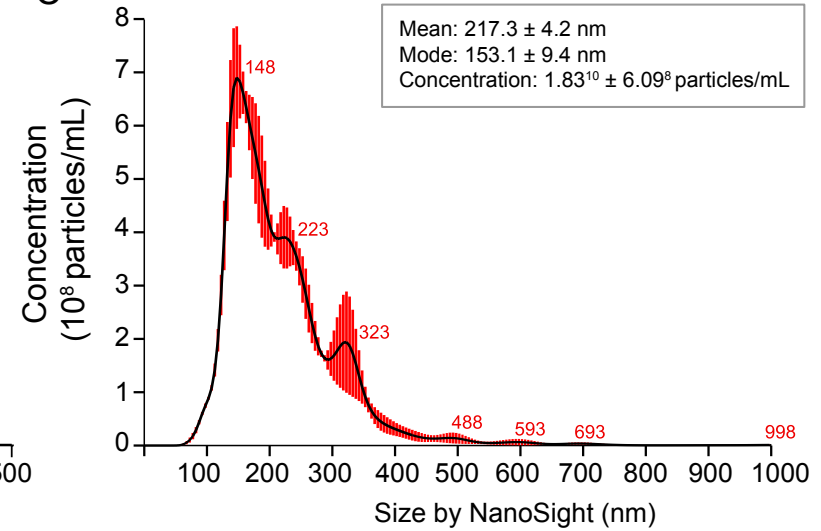

Supplementary Figure 3

Supplement: Supplementary file 3 — Supplementary Figure 3. Size and morphological analysis confirms the presence of extracellular vesicles. A. Cryogenic electron microscopy (cryo‐EM) analysis of extracellular vesicles purified from THP‐1‐derived macrophages infected with Mtb at a MOI of 0.1 shows round vesicles with a distinctive membrane and a diameter and size concurring to extracellular vesicles, as quantified in the following panels. B. Histogram depicting the distribution of the diameter of the extracellular vesicles according to cryo‐EM. C. Histogram showing the size and concentration of extracellular vesicles according to nanoparticle tracking analysis. [file JEV2-10-e12046-s003.pdf]

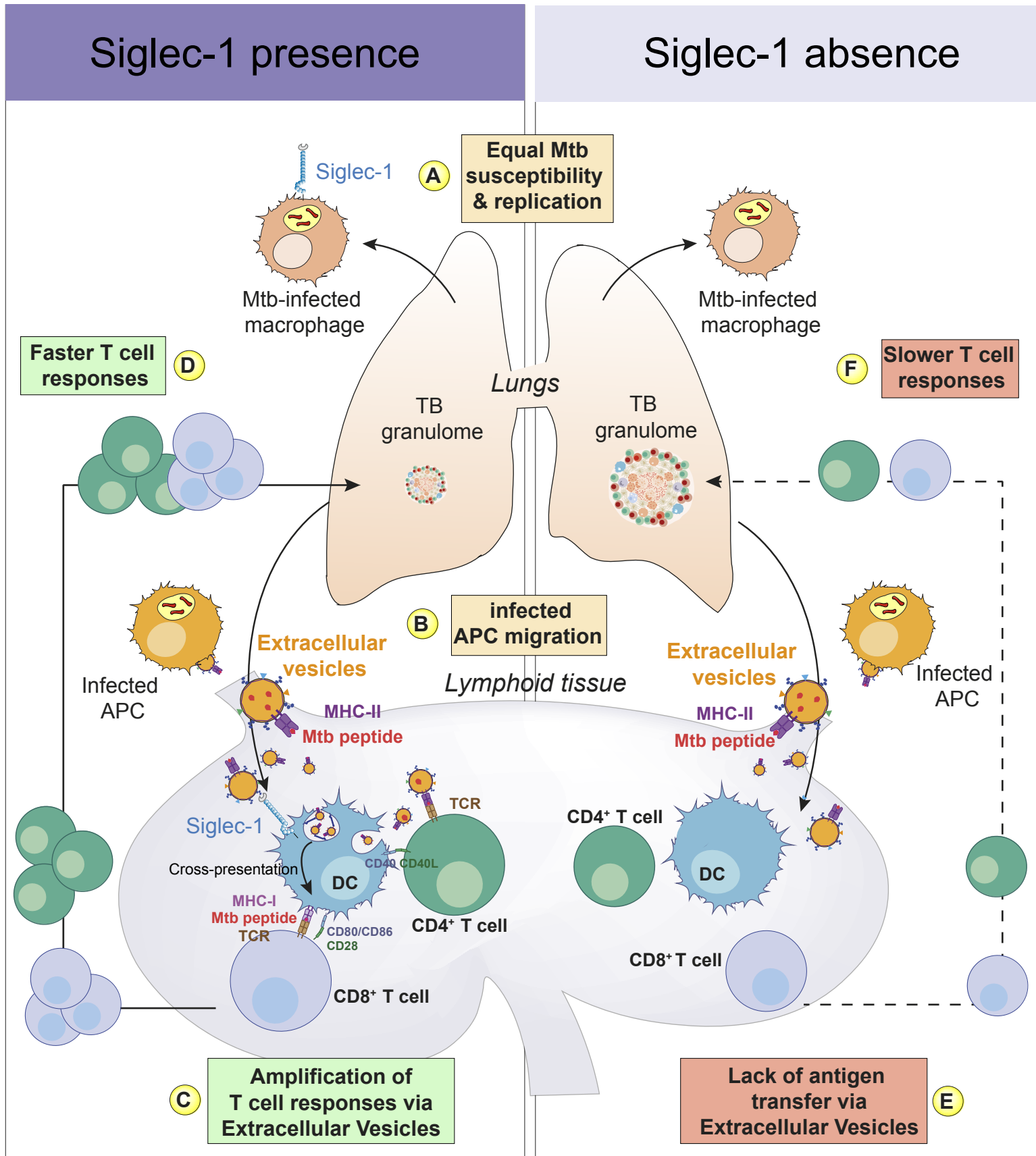

Supplementary Figure 4

Supplement: Supplementary file 4 — Supplementary Figure 4. Hypothetical model explaining the early induction of immunity against Mtb in the presence or in the absence of Siglec‐1. A. Susceptibility to Mtb infection is similar in both cases, as human and mice are equally infected in the absence of Siglec‐1. Moreover, Mtb replication rates are also equivalent in murine models and human cells with varying Siglec‐1 expression levels. B. Once antigen‐presenting cells are infected, these cells migrate to secondary lymphoid tissues, where they are not competent for direct antigen presentation as they are productively infected with Mtb, but can transfer antigens to competent uninfected antigen presenting cells through extracellular vesicle release, which are captured via Siglec‐1. C. Antigen uptake by Siglec‐1 amplifies the initiation of T cell responses. D. These T‐cell responses are mounted faster and contain the pulmonary damage. E. In sharp contrast, the lack of Siglec‐1 compromises antigen exchange via extracellular vesicles. F. Thus, in the absence of Siglec‐1, T‐cell responses are mounted later and are not able to control the early pulmonary damage leading to bacterial dissemination. [file JEV2-10-e12046-s004.pdf]
