# Supplementary material for: Dissemination of Mycobacterium tuberculosis is associated to a SIGLEC1 null variant that limits antigen exchange via trafficking extracellular vesicles
Source: J Extracell Vesicles. 2021 Jan 14;10(3):e12046. doi: 10.1002/jev2.12046 (PMC7807485; doi:10.1002/jev2.12046)
Supplement: Supplementary file 5 — Supplementary information [file JEV2-10-e12046-s005.pdf]

Supplementary table 1. Demographic characteristics of individuals from the TB cohort				
	Controls		TB group	
	Null variant (heterozygous)	Common variant (homozygous)	Null variant (heterozygous)	Common variant (homozygous)
Age median; (IQR)	25.5 (20-35.25)	28 (21-41)	41.5 (32.75-52)	39 (31-50)
Female n; (%)	7 (14.9%)	764 (27%)	12 (21.4%)	592 (24%)
Male n; (%)	40 (85.1%)	2066 (73%)	44 (78.6%)	1876 (76%)
Russian mother n; (%)	44 (93.6%)	2405 (84.9%)	49 (87.5%)	2099 (85%)
Russian father n; (%)	40 (85.1%)	2335 (82.5%)	45 (80.4%)	2024 (82%)

Supplementary Table 1
